# Computational Insights into the Dynamic Structural Features and Binding Characteristics of Recombinase UvsX Compared with RecA

**DOI:** 10.3390/molecules28083363

**Published:** 2023-04-11

**Authors:** Yue Pan, Ningkang Xie, Xin Zhang, Shuo Yang, Shaowu Lv

**Affiliations:** 1 Key Laboratory for Molecular Enzymology and Engineering of the Ministry of Education, School of Life Science, Jilin University, 2699 Qianjin Street, Changchun 130012, China; 2 Bioarchaeology Laboratory, Jilin University, 2699 Qianjin Street, Changchun 130012, China

**Keywords:** DNA recombinases, RecA, UvsX, homologous modelling, molecular dynamics simulations

## Abstract

RecA family recombinases are the core enzymes in the process of homologous recombination, and their normal operation ensures the stability of the genome and the healthy development of organisms. The UvsX protein from bacteriophage T4 is a member of the RecA family recombinases and plays a central role in T4 phage DNA repair and replication, which provides an important model for the biochemistry and genetics of DNA metabolism. UvsX shares a high degree of structural similarity and function with RecA, which is the most deeply studied member of the RecA family. However, the detailed molecular mechanism of UvsX has not been resolved. In this study, a comprehensive all-atom molecular dynamics simulation of the UvsX protein dimer complex was carried out in order to investigate the conformational and binding properties of UvsX in combination with ATP and DNA, and the simulation of RecA was synchronized with the property comparison learning for UvsX. This study confirmed the highly conserved molecular structure characteristics and catalytic centers of RecA and UvsX, and also discovered differences in regional conformation, volatility and the ability to bind DNA between the two proteins at different temperatures, which would be helpful for the subsequent understanding and application of related recombinases.

## 1. Introduction

Homologous recombination is the key step of DNA metabolism and a conserved biological process [1,2]. The RecA recombinase family proteins are used to catalyze DNA strand exchange reactions, which are central to homologous recombination and recombinant DNA repair mechanisms. Their functional and catalytic abilities are focused on (i) binding to a single-stranded DNA (ssDNA) and aligning to a homologous sequence in another double-stranded DNA (dsDNA) and (ii) transfer of a strand of DNA from the double-stranded to the initial ssDNA to form a stable heterogeneous DNA duplex [1]. This family includes RecA in prokaryotic cells, Rad51 in eukaryotic cells, and UvsX encoded by Phage T4; these play a crucial role in DNA management by playing a role in repair, recombination and maintenance of genome stability [3,4,5]. Knockout of RecA in bacteria results in the accumulation of harmful mutations leading to cell death [6]. Similarly, Rad51 knockout mice showed cell death and embryo non-survival [7]. Therefore, RecA family proteins are of fundamental importance to cellular vitality in all domains of life. The RecA protein of *E. coli* is the prototype of this kind of protein and has received the most in-depth study; its functional characteristics and structure have been comprehensively studied [8,9,10].

Bacteriophage T4 is an important system for studying recombination and DNA metabolism [11,12,13]. T4 DNA replication also occurs primarily through a recombination dependent mechanism. UvsX protein is the recombinant enzyme encoded by bacteriophage T4 and belongs to the RecA recombinase family [14,15,16]. It is a 40 kDa protein with a function similar to that of the RecA protein in *E. coli*. It polymerizes nonspecifically on single-stranded DNA (ssDNA) to form helical filaments that later bind to homologous double-stranded DNA (dsDNA), and the subsequent 5′ to 3′ branch migration forms heterogeneous dsDNA. Since recombination intermediates initiate phage DNA replication late in infection, UvsX is also implicated in recombination dependent DNA repair pathways. Therefore, UvsX has a variety of functions, in the study of recombinant mechanisms and more generally [17,18].

UvsX has 28% sequence similarity with *E. coli* RecA and 51% sequence similarity with its catalytic core domain; moreover, the protein structure of UvsX is highly consistent with that of RecA [1,19]. Its monomer consists of a large core region and two small regions of N-terminal and C-terminal [20]. The N-terminal and C-terminal domains are mainly involved in the aggregation of molecules into filaments. The core domain contains a mixed eight-stranded β-sheet franked by two α-helices on one side and by two more α-helices on the other side. The overall folding of UvsX is very similar to that of RecA despite the relatively poor sequence conservation of UvsX and RecA. The two monomers in an asymmetric unit are formed by two interacting surfaces, one on the C-terminal and the other on a surface on the core domain, consisting mainly of residues Phe65, Arg79, Tyr99 and Met103. ATP binds within the interacting surface on the core domain, and the ATP-binding sites of UvsX are conserved both in terms of location and residue composition. The two DNA binding loops L1 and L2 are also part of the core domain. L1 and L2 of UvsX are disordered in structures such as RecA and each monomer binds three nucleotide or DNA base pairs [19,21]. Although the structural basis of homologous chain pairing and exchange is well understood in biochemistry, it has not been fully elucidated. Furthermore, the structural, biochemical and biophysical analyses of UvsX have been reported, but the molecular dynamics of UvsX have not been developed. In this study, we focused on UvsX protein binding of both ATP and dsDNA, and used RecA as a reference. The following four systems were designed: 298 K RecA, 310 K RecA, 298 K UvsX, and 310 K UvsX. Molecular and dynamic descriptions of RecA and UvsX properties indicate that, although the sequence consistency of RecA and UvsX is low, they are highly conserved in their conformation, as well as in their catalytic characteristics involving ATP and their effects on DNA base binding. However, these two proteins have their own characteristics in terms of correlation between domains, DNA binding region fluctuations and binding properties at different temperatures. This study seeks to provide new insights into the conservation and variability of these two recombinant enzymes.

## 2. Results

### 2.1. Sequence Alignment and Initial Models

**Figure 1:**
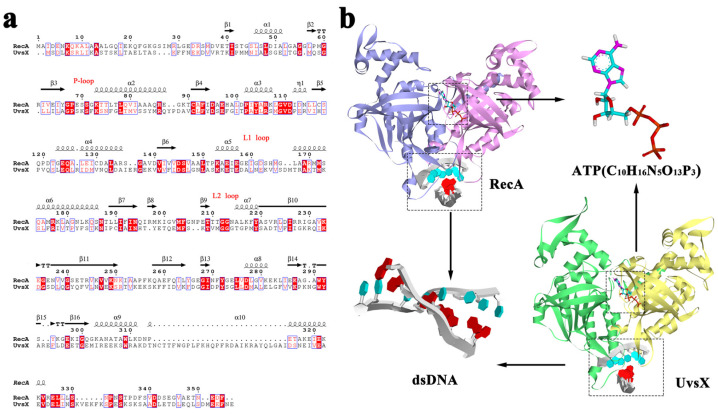
(**a**) Sequence alignment of RecA and UvsX. Regions with the same amino acid sequence are filled with red background, and regions with similar amino acids are filled with red letters. (**b**) Overview of initial structures included simulated initial structure of RecA, ATP structure, dsDNA structure and simulated initial structure of UvsX.

*Escherichia coli* (*E. coli*) RecA protein and Bacteriophage T4 UvsX protein are both members of the RecA recombinase family. In the comparison of amino acid sequences of the two proteins, the sequence identity is 23.55% (Figure 1a) and many conserved residues are located around ATP-binding sites and in the hydrophobic interior of proteins. Walker A motif or ‘P-loop’ is an important conserved domain in ATP binding sites. The P-loop of RecA contains residues 65 to 73, which is equivalent to residues 59 to 67 of UvsX. The ATP binding site is located at the interface between monomers (Figure 1b), and residues 248–254 of adjacent monomers provide an enclosed platform for ATP, where amino acids are also conserved. The two conserved crucial residues fixing ATP phosphate groups are Lys248, Lys250 in RecA. In UvsX, there are two residues with the same function including polar amino acids, Lys246 and Arg248. The conservation surrounding the active site of the ATPase is appropriate because both substrate binding and catalysis require the presence and precise positioning of functional groups. In RecA, the crucial DNA binding domains are the L1 loop and L2 loop with residues 157–164 and 195–209, and the corresponding positions in UvsX are residues 152–165 and 198–211. The DNA-binding region is not highly conserved, and there is no obvious pattern of positively charged residues. It is possible that DNA is bound by contact with the backbone of the polypeptide. Despite the relatively poor sequence conservation, in the structure of crystal resolution, RecA and UvsX are very similar in overall architecture and fold, as well as in the helical structure formed on DNA. In our simulation trajectories, the 3D conformations of each conserved domain of RecA and UvsX were also conserved [19].

The initial structure of RecA used for simulation was based on the first and second monomers of PDB 3cmx (Figure 1b). The original ligand ADP-AlF4 was replaced with ATP, and the DNA double-stranded structure was retained by seven pairs of bases. Many segments of the structure were omitted in the crystal structure of UvsX (PDB ID: 3io5), including the DNA-binding region, and the correct structure of the filament that binds DNA is currently lacking. A multi-template homologous modeling method was adopted in this study. We used the UvsX structure (PDB ID: 3io5) and the RecA structure (PDB ID: 3cmx) with ATP substituted by ADP-AlF4 as the template. Referring to the filamentous formation of RecA protein on dsDNA, loop refining and binding of ATP and dsDNA at their respective sites, the UvsX protein dimer shown in Figure 1b was constructed. The Ramachandran diagram of the UvsX protein (Figure S2) shows that the proportion of amino acid residues in the most favoured regions is more than 90% of the whole protein, so the conformation of this model can be considered to conform to the rules of stereochemistry. As protein dimers were the initial structures utilized in the simulations, the pictures have been labeled with the letters A for monomer I and B for monomer II to distinguish between them.

### 2.2. Analysis of Protein Characteristics

Four systems were set up: 298 K RecA, 310 K RecA, 298 K UvsX, and 310 K UvsX, with each system subjected to three independent 200 ns dynamic simulations. The RMSD curves are similar (Figure S3), indicating that the simulation results can be generalized. All the curves tended to be stable in the later parts of the simulation, indicating that the system was nearly stable and the trajectories could be used for subsequent analysis.

The radius of gyration (Rg) is considered as an indicator of protein conformation densification (Figure S4). The Rg values of the same proteins at the two studied temperatures were not significantly different, but the Rg values of the UvsX protein were higher than those of RecA, indicating that the conformation of the UvsX protein was looser. The solvent accessible surface area (SASA) is used to describe the degree of protein exposure in the solvent (Figure S5). The SASA values of the same protein did not differ significantly at the two temperatures; the SASA values of UvsX protein were higher than those of RecA protein, indicating that UvsX was more exposed to the solvent than RecA. These results indicate that UvsX has a looser conformation and greater exposure to solvent than RecA in the state of bindingATP and dsDNA.

**Figure 2:**
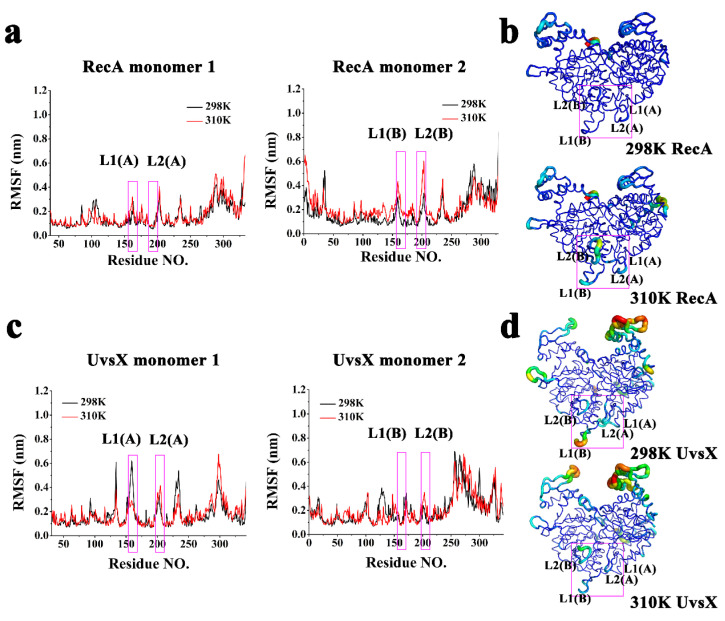
(**a**) RMSF curves of RecA monomers; (**b**) Visualizations of the backbone flexibility of RecA; (**c**) RMSF curves of UvsX monomers; (**d**) Visualizations of the backbone flexibility of UvsX. (The DNA binding domains were framed with pink rectangles.)

RMSF values of the proteins were also calculated to analyze the fluctuation of each residue of the protein (Figure 2a,c). RecA and UvsX showed similar fluctuations in most residues at both 298 K and 310 K. The differences were mainly distributed at L1 and L2 which are the DNA binding domains. For RecA protein, the fluctuation of L2 of monomer I and of L1 and L2 of monomer II are enhanced at 310 K. For the UvsX protein, the L1 region of monomer I showed enhanced fluctuation at 298 K, while the L2 regions of two monomers showed slight enhancement at 310 K. To observe the fluctuation differences more visually, when mapping the intensity of the fluctuations onto the protein structure, the regions with strong fluctuations are shown as thicker and the color depictions transition to warmer colors. It can be visualized that the volatility of the DNA-binding region of RecA is generally stronger at 310 K, while the volatility of the DNA-binding region of UvsX is generally stronger at 298 K. The reason for this discrepancy might be discovered in the subsequent detailed conformation diagrams.

**Figure 3:**
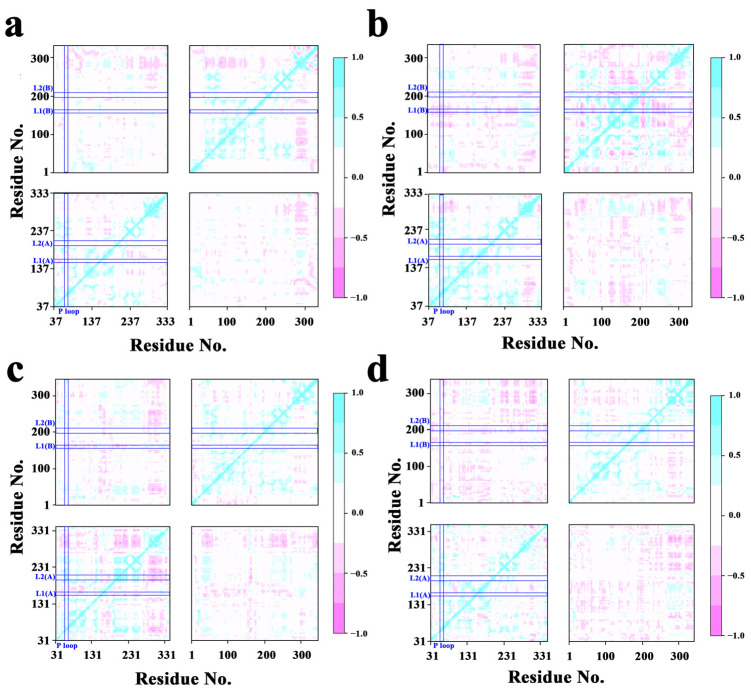
Cross-correlation matrices between residues. (**a**) 298 K RecA; (**b**) 310 K RecA; (**c**) 298 K UvsX; (**d**) 310 K UvsX.

To determine the properties and activities of the proteins, cross-correlation analysis was used to examine protein correlations (Figure 3). For the RecA protein, the correlation between residues was greater at 310 K compared to 298 K, including the correlation between monomers. The correlation between monomers for the UvsX protein was similarly greater at 310 K. Concentrating on the relationship between the ATP-binding crucial region P-loop and the DNA-binding critical regions L1 and L2, at 310 K, the negative connection between P-loop and monomer II L1 in the RecA protein system was strengthened. At 310 K, the negative association between the P-loop and L1 of monomer I and L1 of monomer II was decreased in the UvsX protein system.

**Figure 4:**
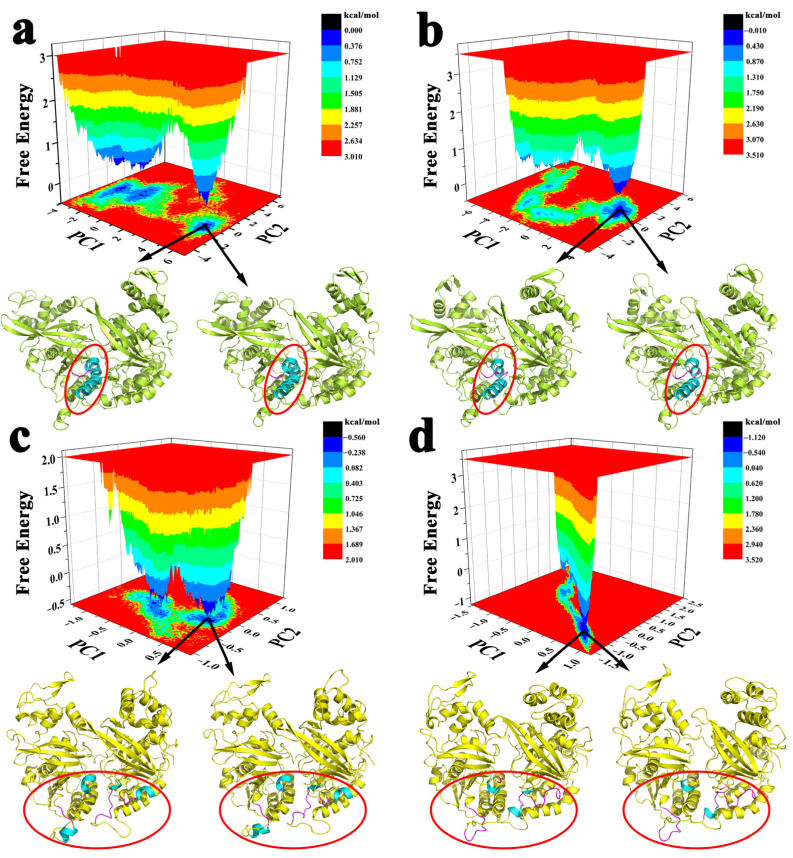
Free energy landscape and lowest energy conformations (**a**) 298 K RecA; (**b**) 310 K RecA; (**c**) 298 K UvsX; (**d**) 310 K UvsX. Comparing the lowest energy conformation of each protein at 298 K and 310 K, the regions with obvious differences are circled in red, and these parts of the protein are colored by secondary structure, with cyan as helix and the magentas as loop.

The FEL analysis was to explore the conformational distribution and stability of the protein from the simulated trajectories. The structural stability was measured based on relative energy minima, where the lower free energy signifies the stronger stability (Figure 4). For RecA protein, at 298 K, the protein would go through two deep energy wells and finally reach the lowest energy conformation, indicating that the deep energy barrier needed to be overcome before reaching the final stable conformation. At 310 K, it did not have to go through such an obvious energy well to get to the lowest conformation. The same was true for UvsX protein, which was more likely to reach a stable conformation at 310 K and needed to cross a deeper basin to become stable at 298 K. The two lowest energy conformations in each simulated trajectory were also extracted. From the conformation diagram of RecA, we found that the helical unwinding occurred at residues 179, 180 and 214 of monomer II at 310 K. For UvsX protein, the helical unwinding occurred at residues 151–155 of monomer I at 298 K, and the helical unwinding occurred at residues 212–216 of monomer I, 151–155 and 216–217 of monomer II at 310 K. The above regions with helical changes are all around L1 and L2, which would affect the binding of proteins and DNA.

### 2.3. Binding Characteristics of Proteins to ATP and dsDNA

The binding properties of RecA and UvsX to ATP or DNA were explored and their binding free energies to ATP or DNA during the entire simulation process were calculated (Figures S6 and S7). The RecA protein has a lower binding free energy to ATP at 310 K, and the same phenomenon applies to the UvsX protein, indicating that both proteins have an increased ability to bind ATP at 310 K compared to 298 K. However, the RecA protein has a high free energy of binding to DNA at 310 K, but UvsX has a low free energy of binding to DNA. UvsX exhibits an unusually unstable binding to DNA when it is at 298 K, while it shows an almost constant binding to DNA at 310 K.

**Figure 5:**
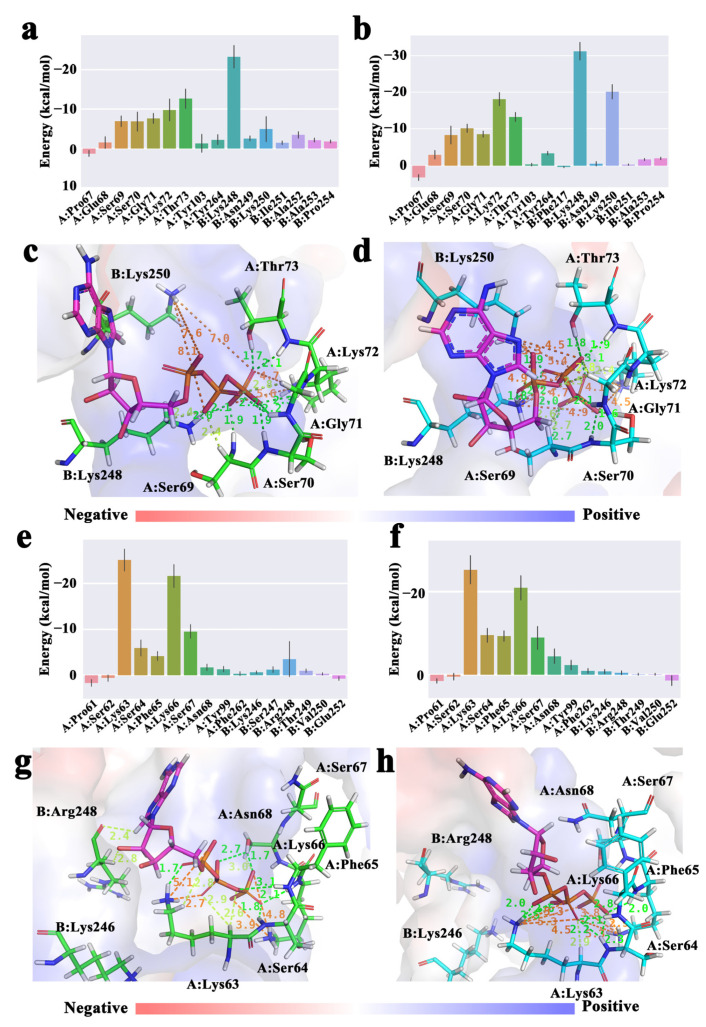
Energy contribution of ATP binding residues in RecA (**a**) 298 K, (**b**) 310 K. Schematic diagram of ATP interacting with RecA (**c**) 298 K, (**d**) 310 K. Energy contribution of ATP binding residues in UvsX (**e**) 298 K, (**f**) 310 K; Schematic diagram of ATP interacting with UvsX (**g**) at 298 K, (**h**) at 310 K. (The dashed green lines represent conventional hydrogen bond interactions, the lemon lines represent carbon hydrogen bonds, and the orange lines represent electrostatic interactions and bond lengths in Å.).

To further capture the energy distribution characteristics and key binding residues of protein to ATP and DNA, the energy component of each ligand-related binding residue was obtained. In the calculation results of RecA protein (Figure 5a,b), residues that mainly play the role of ATP binding function are Ser69-Thr73 of monomer I, Lys248 and Lys250 of monomer II, etc. Pro69-Thr73 of monomer I belongs to the P-loop region of RecA; Tyr248 and Lys250 of monomer II are the aforementioned lysine fingers. ATP was in a positive hole. The conformation of the above important residues interacting with ATP are also shown (Figure 5c,d). At 310 K, the binding energy contributions of Ser70, Lys72, Lys248 and Lys250 were significantly enhanced, the overall bond number was increased, and the bond lengths were shorter, suggesting that these residues are responsible for the better binding of ATP by RecA at 310 K.

In the calculation results of UvsX protein (Figure 5e,f), the residues that mainly play the function of binding ATP in UvsX and the key residues in RecA protein are conserved, which are Lys63-Ser67 of monomer I, Arg248 and Val250 of monomer II, etc. Lys63-Ser67 of the monomer I belongs to the P-loop region of UvsX, and Lys246 and Arg248 of the monomer II correspond to the two lysine fingers of RecA. The conformation of the above important residues interacting with ATP also shown (Figure 5g,h). At 310 K, the energy contributions of Ser64 and Phe65 were significantly enhanced and the P-loop was closer to the ATP, which would prompt the lower free energy of ATP binding to UvsX at 310 K. The results show that both recombinant enzymes bind more strongly to ATP at 310 K, which helps the protein to exhibit ATPase activity.

**Figure 6:**
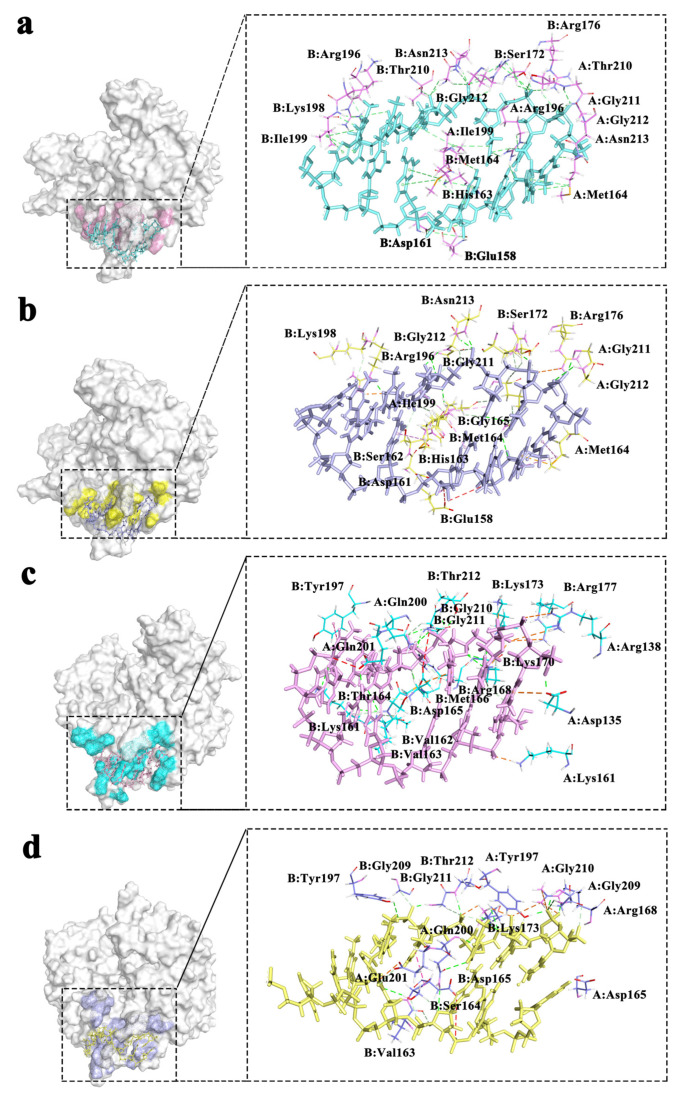
Schematic diagram of DNA binding region (**a**) 298 K RecA; (**b**) 310 K RecA; (**c**) 298 K UvsX; (**d**) 310 K UvsX. (The colors of the dashed lines represent the interactions as shown in Figure 5).

The residues interacting with DNA are mainly concentrated in loop1 and loop2. A detailed diagram of residue interactions is shown in Figure 6, which shows the specific position of each residue’s interaction with DNA, where six pairs of bases are retained. For the RecA complex system, the main binding roles are played by Ile199 of the monomer I and II, Met164 and Arg169 of the monomer II, etc. For the UvsX complex system, the main binding residues are Thr200 of monomer I, Ser164 and Met166 of monomer II. Although the DNA-binding region of the two proteins is not conserved in composition, both have positively charged residues and a residues skeleton that interacts with DNA to maintain a stable binding state to DNA.

### 2.4. DNA Rise Parameters and Communication between ATP and DNA Binding Regions

**Figure 7:**
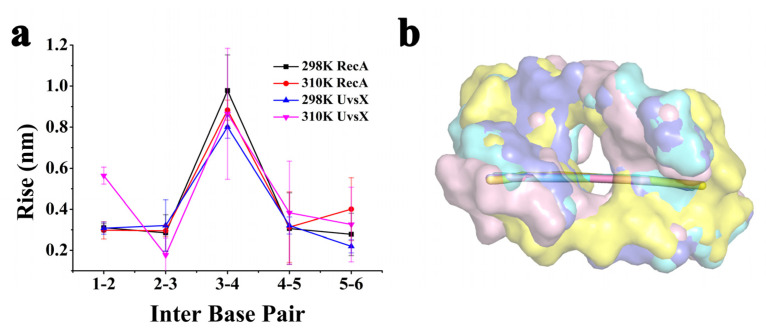
(**a**) DNA rise parameter; (**b**) Diagram of DNA in four systems and the helical axis of the systems. (Cyan for 298 K RecA, purple for 310 K RecA, pink for 298 K UvsX and yellow for 310 K UvsX).

The interaction between protein and DNA is also reflected in the morphological parameters of DNA, and experiments have shown that DNA recombinase stretches DNA strands, so we calculated the rise of successive base pairs used curves+ (Figure 7) [22]. The results show that the translation between the third and fourth positions of the DNA strand is significantly larger than the other sites, and the occurrence of this phenomenon is also a clear confirmation that each monomer binds three base pairs and consequently produces a larger rise between the third and fourth positions [21].

**Figure 8:**
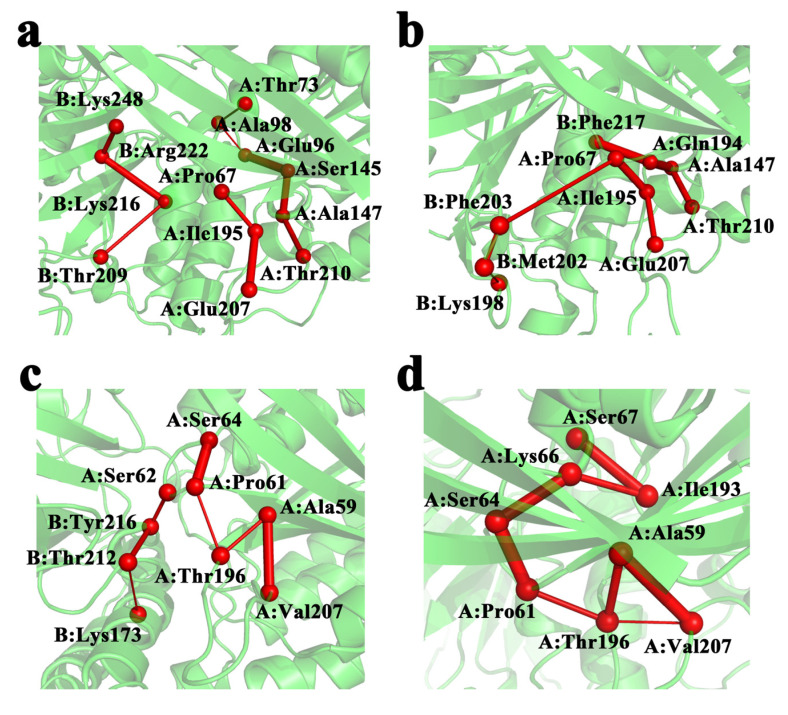
ATP-DNA communication pathway. (**a**) 298 K RecA; (**b**) 310 K RecA; (**c**) 298 K UvsX; (**d**) 310 K UvsX.

The communication between ATP and DNA was also explored by applying the principles of graph theory. We used the ATP-binding region or the DNA-binding region as a collection of residues at the starting and ending points to find communication pathways that could connect the ATP and DNA-binding domains (Figure 8). The Cα of each residue is represented by a small ball, the thickness of the connection between the residue is proportional to the length of the retention time in the simulation, and the first residue in the set is used as the endpoint of the path. In RecA, the shortest path observed was Pro67-Ile195-Glu207 of monomer I, and in UvsX, the most significant link residue was Thr196 of monomer I.

UvsX is similar to RecA in general conformation and reaction mechanism, but there are obvious differences between UvsX and RecA in motion correlation, conformation and dynamics in some parts. This difference may be closely related to UvsX’s unique features.

## 3. Discussion

Homologous recombination as an important process of stable biological genes. The functioning of its core protein of DNA recombinase is closely inter-related with the normal development of living organisms [3,4,23]. Among the RecA family of DNA recombinases, UvsX has not been extensively studied, but the system is simple and widely used. RecA is one of the earliest and most intensively studied. In this study, molecular dynamics simulation of DNA recombinase UvsX was performed, and RecA simulation was performed simultaneously as a comparative study.

Both proteins come from the same family and are highly identical in structure, so their conservation has been proved in many aspects of this study [19]. From the perspective of the overall conformation of the protein, the correlation between RecA and UvsX monomers will be enhanced at 310 K, which means that the cooperation ability between monomers will be enhanced. Similarly, at 310 K the protein is more likely to reach a stable conformation without crossing a high energy barrier. The ATP-binding sites of the two proteins are highly conserved and have strong similarities from amino acid sequence to residue action. In addition, it is noteworthy that RecA and UvsX have a stronger binding capacity to ATP at 310 K compared to 298 K, which is in accordance with the experimental results. The effect on DNA is also consistent, that is, at positions 3 to 4 of the DNA bases, DNA rises significantly more than at other positions, which is experimental proof that each monomer acts on three base pairs.

The UvsX protein is the most characteristic DNA strand exchange protein after the *E. coli* RecA protein. The mechanism by which the UvsX protein facilitates DNA strand exchange is identical to that of the RecA protein overall, but differs from it in some details. In the analysis of protein Rg and SASA, the values of UvsX were significantly higher than those of RecA, indicating that the protein conformation was loose and exposed in the water environment, forming a more expansive monomer volume on the DNA. This may be related to the average helical pitch of UvsX being larger than that of RecA when the average number of subunits per revolution is nearly equal [24]. It also indicates that the UvsX will be large in size and loose in conformation when performing its functions. This may indicate that UvsX has other capabilities that are different from RecA. Meanwhile, the most noteworthy observation is the performance on dsDNA binding at both temperatures. RecA has a stable free energy of binding to dsDNA as a whole at 298 K and fluctuates at 310 K, while UvsX protein is exceptionally unstable in binding to dsDNA at 298 K but binds well at 310 K. The above results were mapped to the binding contribution of each residue, and it was found that the causes of DNA binding instability were all due to the residue near the first DNA base, except that, in RecA, the instability occurred at 310 K while in UvsX it occurred at 298 K. This result also verifies why the DNA binding region of 298 K RecA shows less fluctuation while the DNA binding region of 310 K RecA shows more fluctuation in the RMSF results, and why the opposite phenomenon is found with UvsX. This result indicates that the two temperatures have an opposite effect on the two proteins binding DNA.

We also demonstrate the communication route connecting the two binding sites in these states. The residues Ile195 and Thr196, corresponding to RecA and UvsX, respectively, were found to act as the link between ATP and DNA domains. Perhaps we can explain our results in terms of ATP linkage to DNA domains. During the formal function of the RecA protein, ATP c-phosphate is sensed by two lysine residues through the RecA-RecA interface, which are called lysine fingers and stimulate ATP hydrolysis [15]. The ATPase activity of RecA prevents the accumulation of toxic complexes caused by RecA binding directly to the undamaged dsDNA region [25]. For RecA, enhanced binding of ATP may result in weaker binding of the protein to dsDNA, both in terms of release of dsDNA after completion of homologous recombination and in terms of misbinding dsDNA. However, experiments with UvsX demonstrated good binding to dsDNA in the absence of ssDNA presence, which may be related to the fact that UvsX is a critical factor in the outbreak of T4 viral infection. From the perspective of correlation between the two regions, the correlation analysis of protein residues obtained is also consistent with this: for RecA, the negative correlation between P-loop region and L1 region of monomer II would be enhanced at 310 K, while UvsX would weaken the negative correlation between P-loop region and L1 region of monomer I and monomer II at 310 K. This can be regarded as a preliminary view, to be further demonstrated.

The above results elucidate to a certain extent the mechanism of how RecA and UvsX proteins interact with ATP and dsDNA molecules simultaneously. This may be of great importance for understanding the robust occurrence of homologous recombination and the activity mode during the relevant metabolic period.

## 4. Materials and Methods

### 4.1. Molecular Docking

AutoDock 4.2.6 was used to dock ATP in RecA and UvsX [26]. The structure of ATP was obtained from ChemSpider “http://www.chemspider.com (accessed on 26 June 2021)”. The structure of RecA was reached at PDB database “https://www.rcsb.org (accessed on 26 June 2021)” and its PDB ID is 3cmx [21]. The structure of UvsX was generated by homologous modeling. The two proteins and ATP structures were converted to PDBQT format using AutoDockTools 1.5.6 [27]. Polar hydrogen atoms and Kollman charges were encompassed in proteins [28]. The active site was determined from the binding site of ADP-AlF4 in the crystal structure of RecA (PDBID: 3cmx), and the binding site of ATP in UvsX was determined by the phosphoric acid binding site of UvsX crystal structure (PDBID: 3io5), which were set as the center of the grids. The size of the grid was set to 25 × 40 × 25 Å and the grid spacing was 0.375 Å. All docking simulations were done using the hybrid Lamarckian genetic algorithm (LGA) [29], with a maximum of 2,500,000 energy; AutoDockTools 1.5.6 was used as starting model for investigation; 100 runs were performed. As a selection criterion, the lowest free energy values of binding were chosen. The interactions between ATP and proteins are shown in Supplementary Materials Figure S1. These conformations were further taken as the starting conformation to carry out the MD simulations as described.

### 4.2. Homology Modeling

Homologous modeling is a computational method to generate the 3D structure of a target protein with reference to a similar protein structure. In the absence of a definite structure of the target protein, we can use computational methods to fill this gap, and use at least one known protein 3D model, namely as a template related to the structure of the target protein, to conduct homology modeling [30,31]. The model can be used to analyze protein function and improve protein activity. MODELLER is a computer program for comparative protein structure modeling based on sequence comparison of target proteins and templates and atomic coordinates of templates [32,33]. In this study, the amino acid sequence of UvsX protein was retrieved from NCBI protein sequence database “https://www.ncbi.nlm.nih.gov (accessed on 26 June 2021)”. The known but partially missing UvsX protein structure (PDB ID: 3io5) and its homologous RecA protein structure (PDB ID: 3cmx) were used as templates [19,21]. The DNA binding regions of the UvsX protein are the L1 and L2; the crystal structure of these parts are missing and flexible. Traditional docking methods make it difficult to determine the exact location of the docking, so we treated it as part of the homology modelling and set constraints on the DNA location. Based on the structural conservation of RecA and UvsX, it can be considered that the DNA binding site is similar. Therefore, the RecA structure template containing DNA (PDB ID: 3cmx) was used to automatically extract and satisfy the distance constraints of the model relying on MODELLER. The selected restriction sites were the four conserved sites of dimeric RecA and UvsX, as Gly211 and Gly212 of RecA, and the corresponding Gly209, Gly210 of UvsX, with detailed IDs in the script as, (‘N:211/209:A’, ‘OP1:DT2’), (‘CA:212/210:A’, ‘O5:DT1’), (‘N:211/209:B’, ‘OP1:DT5’), (‘CA:212/210:B’, ‘O5:DT4’). Next, by optimizing the structure of L1 and L2, the model with the lowest Discrete Optimized Protein Energy (DOPE) value was selected as the initial structure of UvsX. Multi-template modeling, DNA joining and loop optimization were performed using the MODELLER 10.2 advanced program. After ATP docking, the modeled structure was used as the initial structure for subsequent simulations.

### 4.3. Molecular Dynamics Simulations

All atom MD simulations were performed using GROMACS 2021.2 package [34]. Amber14sb_parmbsc1 was used as the force field of the simulations which can more accurately describe protein and DNA [35]. The parameterization of ATP was performed by the acpype, a python interface to Antechamber server [36,37]. The protein was solvated using TIP3P water model in a cubic box with sides of 1.0 nm [38]. The system was neutralized by adding sodium and chloride ions. Particle Mesh Ewald was used for long-range electrostatics with a cutoff of 1.2 nm [39]. The short-range electrostatic interaction cut-off is 1.2 nm. The linear constraint solver (LINCS) algorithm was applied to constrain covalent bonds; the hydrogen atoms were constrained using the SHAKE algorithm [40,41,42]. The first step in the simulation is minimization and the steepest descent minimization algorithm was used. The maximum number of minimization steps to perform was 50,000 and stop minimization was applied when the maximum force < 10.0 kJ/mol. Further, temperature equilibration was performed for 100 ps by coupling to a temperature bath using a Nosé–Hoover thermostat with a coupling time constant. From this step, two protein systems, UvsX and RecA, were simulated at 298 K and 310 K, respectively. The following pressure equilibration and pressure coupling with Berendsen at 1 bar were performed 100 ps. All MD simulations were performed at their own constant temperature and run to 200 ns with 2 fs for timestep. Each system had three independent 200 ns trajectories. Due to the exceedingly unstable nature of the conformation before 50 ns, data analysis except for those presented on a time-scale was performed by combining the three trajectories after 50 ns. Analysis of the simulation trajectories such as root-mean-square-deviation (RMSD), root-mean square-fluctuation (RMSF), Rg, and SASA were carried out using GROMACS package g_rmsd, g_rmsf, g_gyrate, g_sasa, respectively.

### 4.4. Principal Component and Free Energy Landscape Analysis

Principal component analysis (PCA) method, also known as quasi-harmonic analysis or fundamental dynamics method, aims to reduce the dimensions of complex systems. Most fluctuations of the system can be described by a few principal component feature vectors or principal components [43]. Residue correlation analysis was performed based on principal component analysis using Bio3D software [44]. The matrix of all atomic correlations whose elements can be displayed in graphical representation, often referred to as a dynamic cross-correlation map (dccm) was used [45,46]. It can check the internal dynamics of the structure domains. The elements *C_ij_* of DCCMs were calculated by using the coordinates of the Cα atoms. The formula can be expressed as:

*C_ij_* = <(*x_i_*−<*x_i_*>) (*x_j_*−<*x_j_*>)>, (1)

where *x_i_*/*x_j_* is the coordinate of the *i*th/*j*th atom of the systems.

The Energy Landscape Analysis can be represented by the minimum that represents the metastable conformation states of the system and the potential barriers connecting those states. Therefore, an appropriate energy landscape can represent the correct amount of energy and position of metastable and potential barriers, which can be used to understand the stable folding and function of proteins. The formula is:

Δ*G*(*X*) = −*K_B_TlnP*(*X*), (2)

where *K_B_* is the Boltzmann constant, *T* is the temperature of simulation systems and *P*(*X*) is the probability distribution of the molecular system along the PCs.

### 4.5. MM-GBSA Calculations

Molecular mechanics/Generalized-Born Surface Area (MM/GBSA) is one of the most popular methods to estimate binding free energies. This method is accurate and efficient in dealing with proteins and protein nucleic acid systems [47,48]. MM/GBSA is a very general method for estimating binding energies, determining structural stability, predicting hot spots, and evaluating the contribution of individual residues or energy terms through free energy decomposition analysis. The formulas are as follows:

Δ*G_bind_* = Δ*H* − *T*Δ*S* ≈ Δ*E_MM_* + Δ*G_sol_* − *T*Δ*S*, (3)

Δ*E_MM_* = Δ*E_bonded_* + Δ*E_nonbonded_* = (Δ*E_bond_* + Δ*E_angle_* + Δ*E_dihedral_*) + (Δ*E_ele_* + Δ*E_vdW_*), (4)

Δ*G_sol_* = Δ*G_pol_* + Δ*G_non−pol_* = Δ*G_PB/GB_* + Δ*G_non−pol_*, (5)

Δ*G_non−polar_* = *NP_TENSION_* ∗ Δ*SASA* + *NP_OFFSET_*, (6)

In the above equations, Δ*G_bind_* is the free binding energy, Δ*H* is the enthalpy of binding, −*T*Δ*S* is the conformational entropy after ligand binding. Δ*E_MM_* corresponds to the change in molecular mechanical energy in the gas phase. Δ*E_bond_* is known as internal energy, and Δ*E_nonbonded_* corresponds to the van der Waals and electrostatic contributions.

Here we used gmx_MMPBSA program, which is based on AMBER’s mmpbsa.py and uses GROMACS files to perform MM/GBSA calculations [49]. Prior to performing the calculation, 200 snapshots were selected from each MD simulation with a time interval of 1 ns. The Generalized Born (GB) model was used to compute the polar solvation free energies Δ*G_GB_* and the LCPO method was employed to calculate the nonpolar solvation contribution energy Δ*G_SA_*. All residues less than 4 angstroms between the receptor and the ligand were exported as decomposition components of energy.

### 4.6. Protein Structure Network (PSN) and Correlated Path of Communication

The PSN method uses graph formalism to define the network of interacting residues in a given protein from the number of non-covalent interacting atoms.

(7) $$I_{ij}=\frac{n_{ij}}{\sqrt{N_{j}N_{i}}}\times 100$$

where *i* and *j* are residue identifiers, *N_i_* and *N_j_* are normalized values of residues *i* and *j* from the statistically significant protein data set [50].

We used the PyInteraph suite to reveal the paths of long-range structural communication between the two binding region of ATP and DNA [51]. We applied the principles of graph theory, focusing on the structural pattern that can be transmitted from the ATP to the DNA binding site of RecA and UvsX. We considered as interacting pairs any two residues whose side-chain centers of mass lay within 5 Å in RecA and 5.1 Å in UvsX. The cutoff value was produced by the PyInKnife pipeline as a result of calculating the average number and size of the connected components and the hub distribution of a PSN to achieve overlapping ensemble resampling methods, estimating data changes in different resampling [52]. We set cutoff values at 0.1 Å intervals in the 4.9–5.2 Å range, as suggested from other studies [50,53]. In order to avoid forming too sparse or too dense PSNs, we used the number of formed PSNs for comparison (Figures S8 and S9), and retained the network constructed at 5.0 Å cut-off point in RecA and 5.1 Å cut-off point in UvsX for further analysis. We also filtered transient and spurious interactions in the network. This cutoff point was selected based on the size of the maximum connected component found at the different cutoff points, and the optimal criticality rate was determined to be 20% [51].

## Data Availability

Data related to this paper may be requested from the authors.

## Supplementary Materials

The following supporting information can be downloaded at: https://www.mdpi.com/article/10.3390/molecules28083363/s1, Figure S1: The interaction between ATP and protein after docking; Figure S2: Ramachandran diagram of UvsX protein formed by modeling; Figure S3: Root mean square deviation of each system in three 200 ns independent molecular dynamics simulation trajectories; Figure S4: Rg values of each system in three 200 ns independent molecular dynamics simulation trajectories; Figure S5: SASA values of each system in three 200 ns independent molecular dynamics simulation trajectories; Figure S6: ATP binding free energy curves of each system in three 200 ns independent molecular dynamics simulation trajectories; Figure S7: DNA binding free energy curves of each system in three 200 ns independent molecular dynamics simulation trajectories; Figure S8: Number of nodes in the first five connected components at different cut-offs in RecA; Figure S9: Number of nodes in the first five connected components at different cut-offs in UvsX.
