# Supplementary material for: Computational Insights into the Dynamic Structural Features and Binding Characteristics of Recombinase UvsX Compared with RecA
Source: Molecules. 2023 Apr 11;28(8):3363. doi: 10.3390/molecules28083363 (PMC10144138; doi:10.3390/molecules28083363)
Supplement: Supplementary file 1 [file molecules-28-03363-s001.zip › molecules-2290983-supplementary.pdf]

Support Information

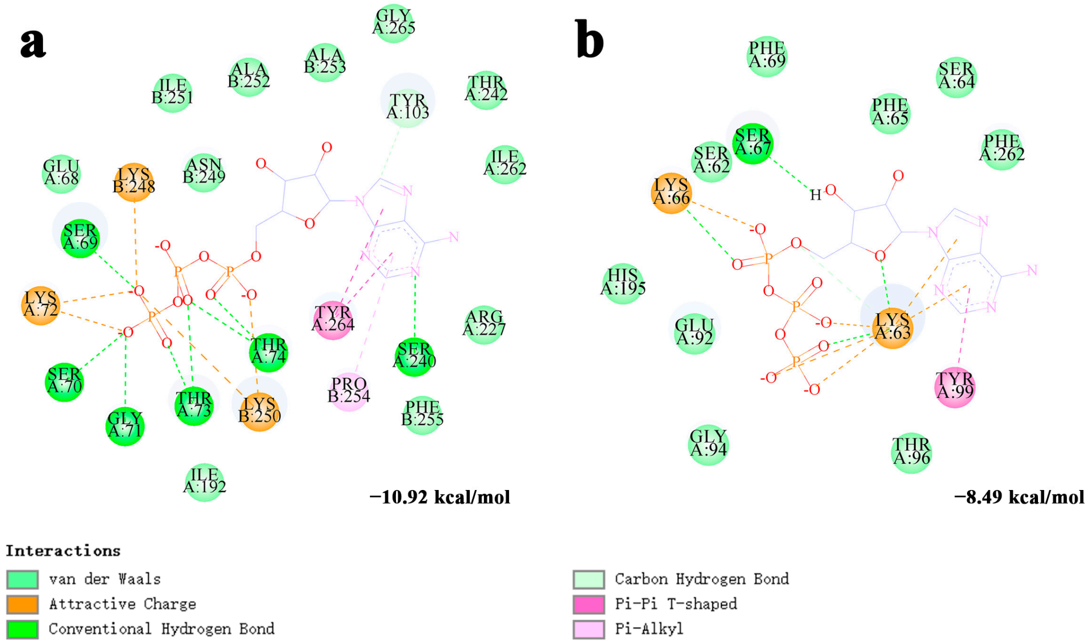

Figure S1. The interaction between ATP and protein after docking. **a.** RecA, **b.** UvsX.

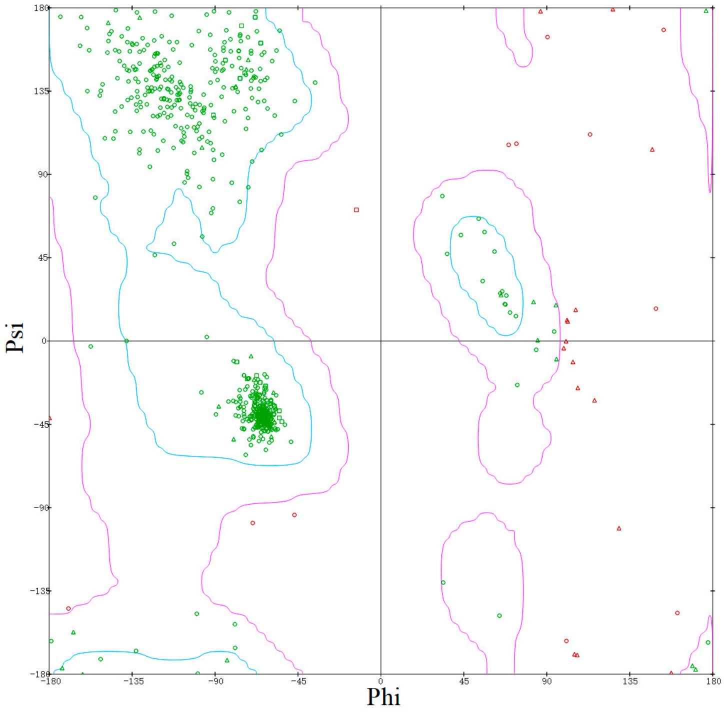

Figure S2. Ramachandran diagram of UvsX protein formed by modeling. The most favoured regions are winded in blue, while the allowed regions are winded in pink.

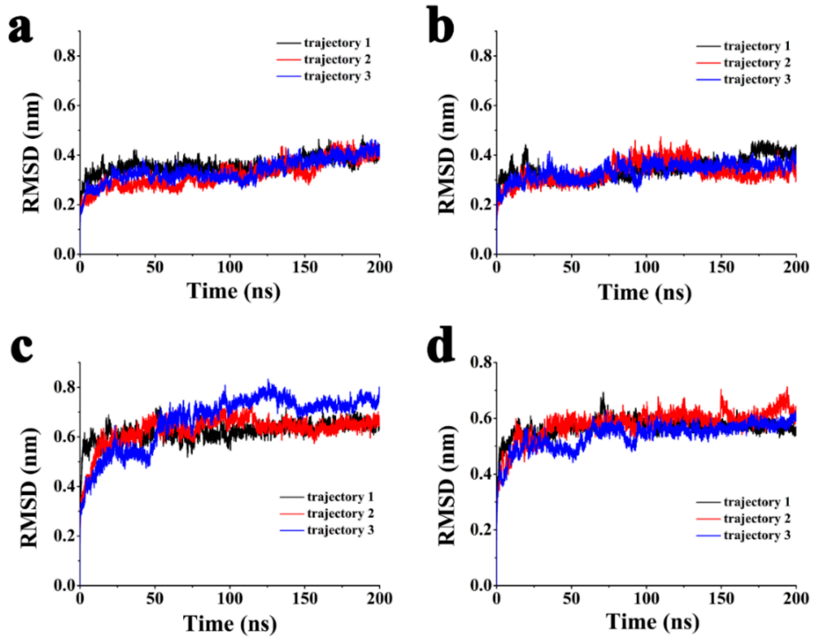

Figure S3. Root mean square deviation of each system in three 200ns independent molecular dynamics simulation trajectories. a. RecA-298K, b. RecA-310K, c. UvsX-298K, d. UvsX-310K.

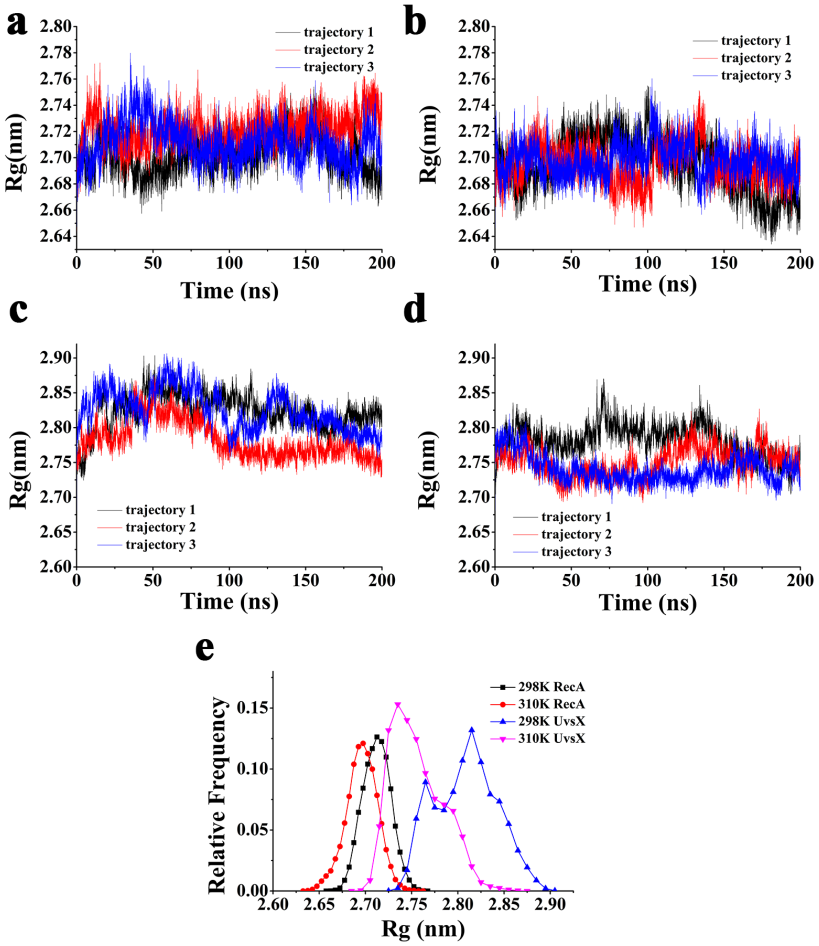

Figure S4. Rg values of each system in three 200ns independent molecular dynamics simulation trajectories. a. RecA-298K, b. RecA-310K, c. UvsX-298K, d. UvsX-310K. f. Relative frequency of Rg values. (The three trajectories after 50ns were combined to calculate.)

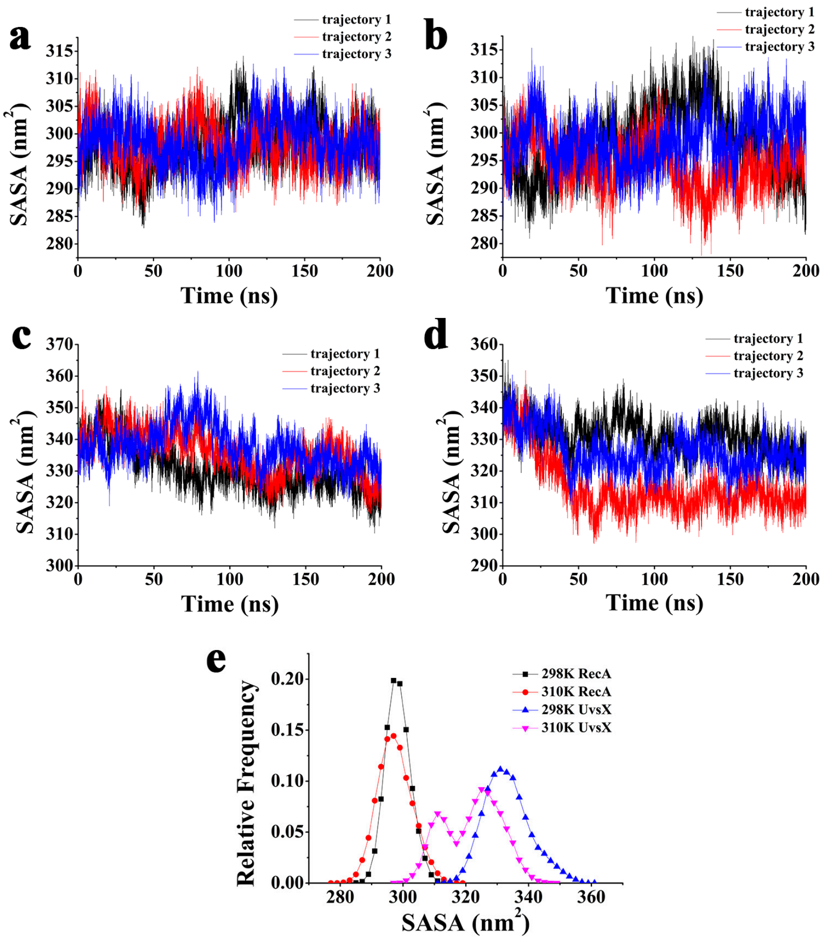

Figure S5. The solvent accessible surface area (SASA) values of each system in three 200ns independent molecular dynamics simulation trajectories. a. RecA-298K, b. RecA-310K, c. UvsX-298K, d. UvsX-310K. f. Relative frequency of SASA values. (The three trajectories after 50ns were combined to calculate.)

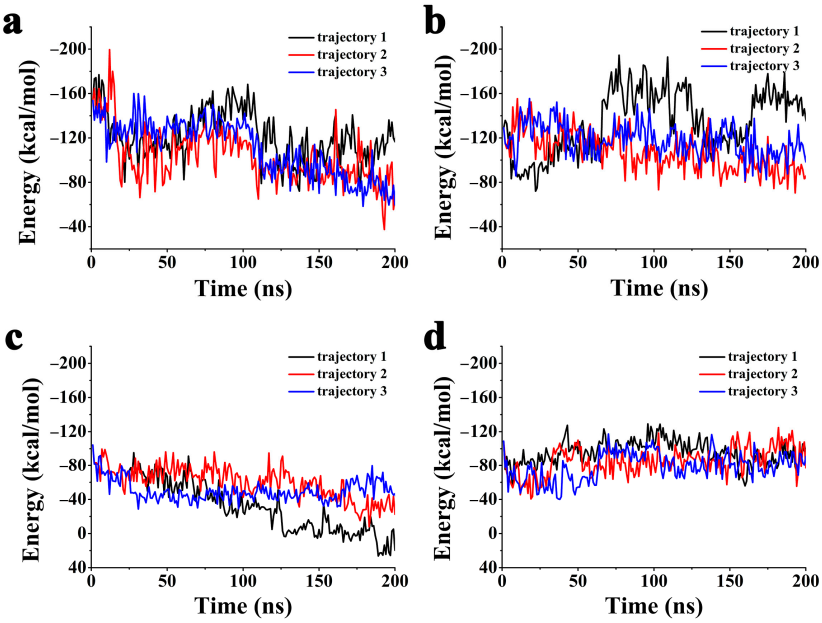

Figure S6. ATP binding free energy curves of each system in three 200ns independent molecular dynamics simulation trajectories. a. RecA-298K, b. RecA-310K, c. UvsX-298K, d. UvsX-310K.

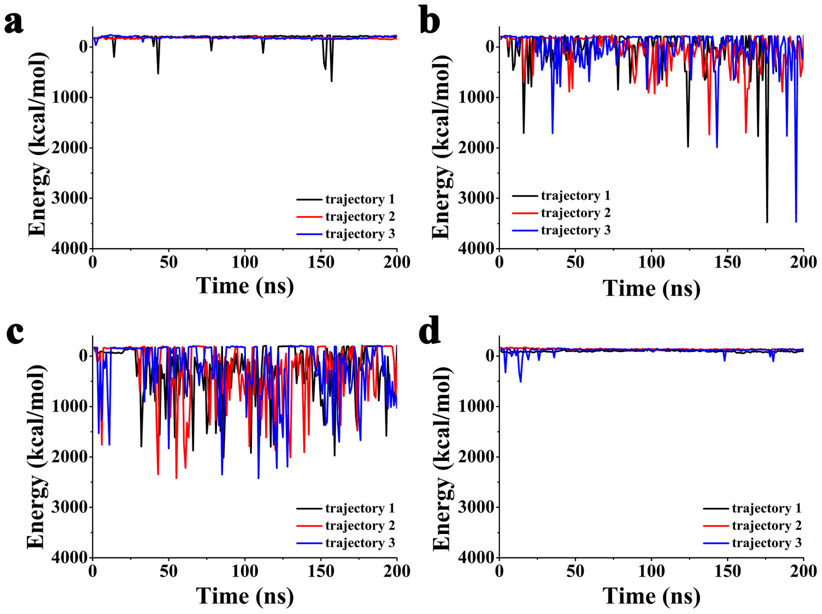

Figure S7. DNA binding free energy curves of each system in three 200ns independent molecular dynamics simulation trajectories. a. RecA-298K, b. RecA-310K, c. UvsX-298K, d. UvsX-310K.

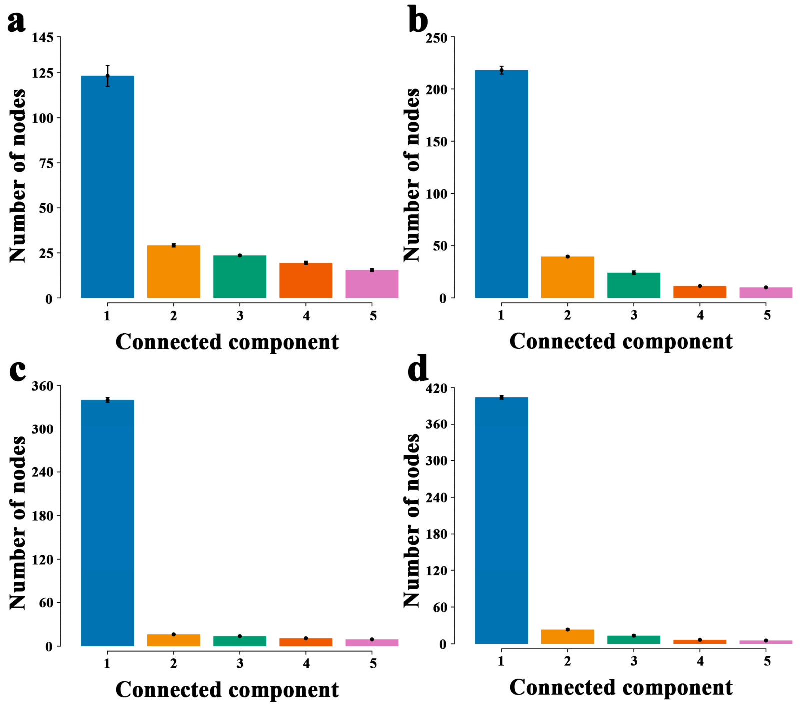

Figure S8. Number of nodes in the first five connected components at different cut-offs in RecA. a. 4.9 Å b. 5.0 Å c. 5.1 Å d. 5.2 Å

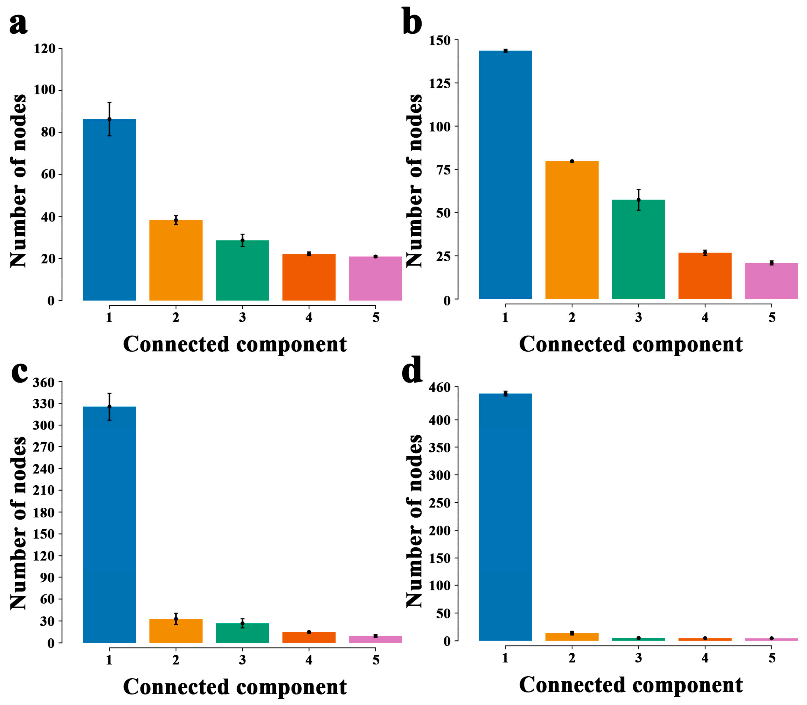

Figure S9. Number of nodes in the first five connected components at different cut-offs in UvsX. a. 4.9 Å b. 5.0 Å c. 5.1 Å d. 5.2 Å
